# Hajj, Umrah, and the neglected tropical diseases

**DOI:** 10.1371/journal.pntd.0006539

**Published:** 2018-08-16

**Authors:** Mashal M. Almutairi, Waleed Saleh Alsalem, Mazen Hassanain, Peter J. Hotez

**Affiliations:** 1 Prince Naif Health Research Center, King Saud University, Riyadh, Saudi Arabia; 2 Department of Pharmacology and Toxicology, College of Pharmacy, King Saud University, Riyadh, Saudi Arabia; 3 Vaccines and Biologics Research Unit, College of Pharmacy, King Saud University, Riyadh, Saudi Arabia; 4 Departments of Pediatrics and Molecular Virology and Microbiology, National School of Tropical Medicine, Baylor College of Medicine, Houston, Texas, United States of America; 5 Center for Neglected Tropical Diseases, National Health Laboratory, Saudi Ministry of Health, Riyadh, Saudi Arabia; 6 Department of Surgery, College of Medicine, King Saud University, Riyadh, Saudi Arabia; 7 James A. Baker III Institute of Public Policy, Rice University, Houston, Texas, United States of America; 8 Scowcroft Institute of International Affairs, Bush School of Government and Public Policy, Texas A&M University, College Station, Texas, United States of America; 9 Department of Biology, Baylor University, Waco, Texas, United States of America; Northeastern University, UNITED STATES

## Introduction

**Fig 1:**
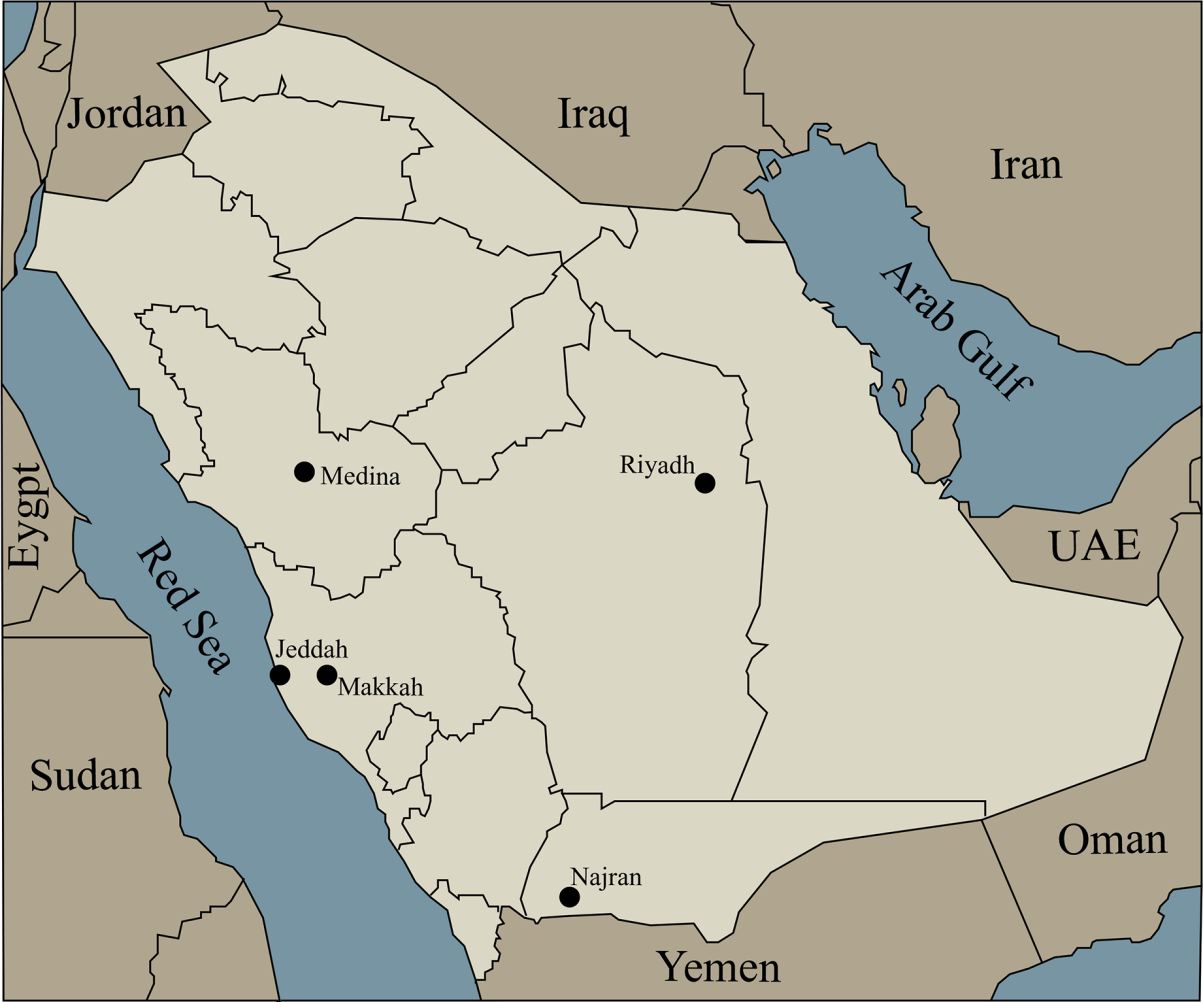
Outline map of Saudi Arabia and surrounding countries. The capital Riyadh, the two holy cities Makkah and Medina, and other cities mentioned in this paper are shown. *Original figure*.

Together, the Hajj and Umrah rank among the leading global venues that host annual mass human migrations. The Hajj is an annual pilgrimage to the Islamic holy city of Makkah in Saudi Arabia (Fig 1). It is considered a religious obligation for all adult Muslims worldwide who have the physical and financial ability and draws an estimated 2–3 million people annually [1]. Umrah is an Islamic pilgrimage to Makkah, which occurs at times other than the period of the Hajj—the period of Ramadan (fasting month) is considered the peak period [2]. In 2018, the Hajj is scheduled to take place in August, while Ramadan will occur between May and June [2].

Through the Hajj and Umrah, it is estimated that visitors to Saudi Arabia arrive from almost every country, based on a ratio of “one pilgrim per 1,000 Muslims from that country” [2]. Currently, the largest countries in terms of Muslim populations are Asian nations located in tropical disease–endemic areas, led by Indonesia, Pakistan, India, and Bangladesh, followed by Nigeria and Egypt in Africa, where neglected tropical diseases (NTDs) are also widespread (Table 1) [3]. Together, these nations account for almost 700,000 Hajj pilgrims, and according to the Global Burden of Disease Study, they account for some of the largest numbers of people living with NTDs [4].

**Table 1:** Countries with the largest numbers of Hajj pilgrims (adapted from [3] and [4]).

Rank [3]	Country [3]	Major NTDs [4]	Number of Hajj pilgrims in 2015 [3]
1	Indonesia	Dengue, Lymphatic Filariasis, Soil-transmitted Helminthiases, Leprosy	168,000
2	Pakistan	Dengue, Trachoma, Kala-azar, Cutaneous Leishmaniasis	143,368
3	India	Dengue, Lymphatic Filariasis, Soil-transmitted Helminthiases, Leprosy, Rabies, Kala-azar, Trachoma	136,020
4	Bangladesh	Dengue, Lymphatic Filariasis, Soil-transmitted Helminthiases, Leprosy, Kala-azar	101,758
5	Nigeria	Schistosomiasis, Intestinal helminth infections, Lymphatic Filariasis, Onchocerciasis, Rabies	76,000
6	Egypt	Schistosomiasis, Intestinal helminth infections	63,000
	Total		688,146

**Abbreviations:** NTDs; neglected tropical diseases

The largest numbers of Hajj immigrants are from South and Southeast Asian tropical countries where globally the largest numbers of cases of dengue, lymphatic filariasis (LF), soil-transmitted helminth infections, leprosy, and kala-azar are also endemic [4]. Similarly, Nigeria is the most highly endemic country in Africa for the major NTDs, especially schistosomiasis, soil-transmitted helminth infections, LF, onchocerciasis, and rabies. Each of these diseases has the potential of being either introduced or reintroduced in the Middle East and North Africa (MENA) region due to Hajj and Umrah activities.

Previously reported major Hajj-associated infectious diseases included respiratory tract infections like seasonal influenza, meningococcal disease, lower respiratory infections due to pneumococcus, and tuberculosis; water-borne and blood-borne infections including hepatitis A, B, and C were discussed elsewhere [5, 6]. In this report, we focus on the major NTDs that have either been introduced into the Middle East through Hajj and Umrah pilgrimages from tropical disease–endemic countries of Asia and Africa or where importation from Saudi Arabia to other parts of the world are possible. In some cases, these diseases have now become endemic in Saudi Arabia and elsewhere in the MENA region. Our report emphasizes the recent scientific literature published within the last five years.

## Arbovirus infections

Because *Aedes aegypti* mosquitoes are found in the western Arabian Peninsula [7], there is potential for arboviral diseases such as dengue, Zika virus infection, yellow fever, and chikungunya to be introduced to Saudi Arabia through Hajj and Umrah activities. This is especially the case when infected individuals, often from Asia, enter Saudi Arabia through Jeddah, the major airport servicing the Hajj and Umrah (Fig 1). The first imported dengue cases from Zanzibar to the Arabian Peninsula were reported in the 19th century, notably in Yemen in 1872 and again in 1877 [8]. It is believed that dengue may have recently emerged through an Asian entry pathway in 1994 when dengue outbreaks were reported and found to be caused by dengue virus-1 (DENV-1) and DENV-2 [5, 7, 9]. Subsequently, DENV-3 may have been introduced in 1997 [9], with all three serotypes still circulating in western Saudi Arabia. Using full genome and envelope (E) gene sequencing of DENV-2 strains in Saudi Arabia, it has been suggested that there continues to be multiple dengue introductions, most recently from the Indian subcontinent, most likely Pakistan, in 2014 [9, 10]. Altogether, there is a high potential that these arboviral diseases could emerge during the Hajj and Umrah.

Based on the ease of introduction of dengue into Saudi Arabia, there are concerns that other *A*. *aegypti* mosquito-transmitted viruses could follow during Hajj seasons. Of particular concern was the possibility of Zika virus infection emerging as a consequence of mass migrations through Jeddah in 2015–2016 [11, 12], although there is no evidence for this actually occurring [10]. Yet another concern was reports of a Chinese worker from Angola introducing the first known case of yellow fever in China in 2016 during the large outbreak across West Africa in that year [13, 14]. This prompted vigilance for yellow fever emerging in western Saudi Arabia either through Hajj and Umrah pilgrimages, or from sub-Saharan Africa across the Red Sea through multiple points of entry [13, 14]. Adding to the worries was the shortage of yellow fever vaccine reported in that year.

Finally, Rift Valley fever (RVF, a *Phlebovirus* in the family Bunyaviridae) is believed to have been introduced at least twice into the MENA region from sub-Saharan Africa including entry into Egypt from Zimbabwe in the 1970s and from Kenya into Saudi Arabia near the border with Yemen in 2000 [15]. The Saudi Arabia–Yemen outbreak is believed to be the first and only epizootic of RVF outside of Africa and may have emerged in part from floods due to heavy rains in that year, which helped to promote transmission through *Culex triteniorynchus* and *Aedes vexans arabiensis* mosquitoes [15–17]. The outbreak affected approximately 40,000 animals, especially sheep and goats, with 883 human cases and 124 deaths in Saudi Arabia, together with 1,328 human cases and 166 deaths in Yemen [17]. Intermittent diagnosis of cases has persisted [17].

## Hemorrhagic fever virus infections

Because ixodid ticks of the genus *Hyalomma* are indigenous to western Saudi Arabia including Makkah city, there is potential for introducing tick-borne hemorrhagic fever viruses [18]. In 1990, a Crimean-Congo hemorrhagic fever virus (CCHF) outbreak happened in Makkah among abattoir workers [18]. The disease was introduced to the western region through the Jeddah seaport via tick-infected imported animals [18, 19].

Another tick-borne (both *Hyalomma* hard ticks and *Ornithodoros* soft ticks) viral infection, Alkhumra hemorrhagic fever virus (AHFV), was first identified in western Saudi Arabia. Between November and December of 1995, AHFV was isolated for the first time from blood samples of six butchers who worked for a slaughterhouse in Alkhumra (a small town near Makkah) [20]. AHFV is considered to be one of the deadliest viral infections, with the case fatality rate ranging between 25%–30% [20, 21]. According to a Saudi Ministry of Health data set, 281 new cases were confirmed during 2011–2014, and all of these cases had clustered in the Makkah and Najran regions (Fig 1) [22]. Comparative analysis between different tick-borne flaviviruses confirmed that AHFV is most closely related to Kyasanur Forest disease virus (KFDV), another hemorrhagic fever virus that causes hemorrhagic fever in Karnataka State, India. Genetic distance mapping further indicates that AHFV is a subtype of KFDV, suggesting that the transmission of AHFV occurred from the Indian subcontinent, possibly through the Hajj and animal importation [21].

Because the 2014 Hajj season coincided with the Ebola outbreak in West Africa, the Saudi authorities suspended issuing Umrah and Hajj visas for people from Guinea, Liberia, and Sierra Leone during that year [23]. Until today, there had been no reported cases of Ebola virus during Hajj. Continuous surveillance against Ebola remains active [24].

## Other viral infection

Since its discovery in 2012, Middle East Respiratory Syndrome Coronavirus (MERS-CoV) infected around 1,800 individuals with a 35% fatality rate. About 80% of the reported cases occurred in Saudi Arabia [25]. The first case of MERS-CoV occurred only a few weeks before the 2012 Hajj season. However, to date, no transmission of MERS-CoV has occurred among Hajj or Umrah pilgrims [2]. In addition, different studies showed no evidence of MERS-CoV transmission among returning pilgrims [2]. Given the fact that a majority of the cases happened in Saudi Arabia, MERS-CoV exportation from people leaving Saudi Arabia during the Hajj or Umrah remains a concern.

## Vector-borne protozoan infections

Saudi Arabia is ranked among the highly endemic countries of cutaneous leishmaniasis (CL). During the last 10 years, the number of reported CL cases has dramatically dropped, with the average reported annual prevalent cases being around 2,500 [26, 27]. Both of the major parasites causing CL in the Middle East, *Leshmania major* (zoonotic species) and *L*. *tropica* (anthroponotic species), and the vector sand flies (*Phlebotomus papatasi* and *P*. *sergenti*) are distributed throughout Saudi Arabia, including the two holy cities (Makkah and Medina). As a result, CL could be potentially transmitted to other countries through Hajj and Umrah pilgrims leaving Saudi Arabia. In fact, different reports indicate that CL may have been introduced to Asia and Europe through Saudi Arabian visitors [28–30]. Compounding this issue is the finding that CL is now hyperendemic in conflict regions in Syria, Iraq, and Yemen [31, 32].

Malaria previously posed a threat to Hajj and Umrah pilgrims in western Saudi Arabia. However, decades of malaria control programs have helped to minimize these threats. For example, between 2010 and 2015, the indigenous malaria cases remained below 100 per year. This number increased to 272 cases in 2016. The total confirmed malaria cases in 2016 was 5,382 (indigenous and imported). The vast majority of these cases (95%) was due to importation, possibly through Hajj, workers and illegal migrants from South Asian and African nations and Yemen, where malaria is endemic [33]. Therefore, beyond the modest levels of autochthonous malaria transmission, Saudi Arabia faces regular importations of malaria and its *Anopheles* mosquito vector, especially from highly endemic African Muslim–majority nations. Surveillance programs in place in Makkah help to limit local transmission [34, 35].

## Enteric infections

The Hajj gathering over a small area of land provides opportunities for the emergence of enteric bacterial infections, which include antibacterial-resistant strains. For example, during the Hajj seasons between 2011–2013, *Salmonella*, *Shigella*, and *Escherichia coli* accounted for the majority of the circulating enteric infections, with approximately more than one third of these bacteria harboring β-lactamases resistance genes [36]. Active acquisitions of different resistant genes were observed among returning pilgrims, including genes resistant to the last resort antibiotic (colistin) for Gram-negative bacteria [37]. Therefore, returning pilgrims could facilitate dissemination of the multidrug-resistant (MDR) bacteria globally. Since MDR bacteria are emerging worldwide, it might be expected that MDR bacteria will become an increasing problem during coming Hajj seasons. The situation is complicated by the lack of vaccines against most bacterial infections combined with the severe decrease in the new antimicrobials in the clinical development pipeline.

Multiple cholera outbreaks occurred during the Hajj between 1877 and 1912. The largest cholera outbreak occurred in 1893 with total deaths of 32,994 at the end of the Hajj season [38]. Cholera during the Hajj was eventually brought under control through several control measures, including quarantine, vaccination, and improvements to infrastructures for surveillance and rapid detection. One concern is the current cholera epidemic in Yemen, where the prevalence is estimated at 266 per 10,000 population. In 2016, 19,500 Yemenis obtained a visa for the Hajj, so it is possible that cholera cases would spread into Saudi Arabia through Hajj and Umrah pilgrimages. The finding that about 80% of individuals infected with *Vibrio cholerae* do not show symptoms and remain undetected complicates detection and surveillance efforts [39].

## Disease control

The government of Saudi Arabia undertakes enormous efforts for planning the Hajj and Umrah seasons each year through 24 different committees representing the different Saudi sectors. The design of the annual plan is coordinated with different international agencies, including the World Health Organization (WHO), the European Centre for Disease Prevention and Control (ECDC), and the United States Centers for Disease Control and Prevention (CDC), which provide more information about development of outbreaks and emerging diseases including NTDs. Based on these combined efforts, the Saudi Ministry of Health issues recommendations for the required vaccinations and other preventive measures.

The ambitious Saudi Arabia’s Vision 2030, “@SaudiVision2030,” (a methodology and roadmap for economic and developmental transformation in Saudi Arabia) includes programs that introduce major transformation to the different sectors and services within the country. A key strategic program is ensuring opportunities to expand safe Hajj and Umrah pilgrimages [40]. To achieve this goal, the Saudi government recently started expansion plans for the two holy mosques in Makkah and Medina as well as efforts to increase the capacities of the international and regional airports in and around the two holy cities. In 2015, the number of Umrah pilgrims was 8 million, but this number is expected to reach 18 and 30 million by 2020 and 2030, respectively.

As a result of the increase in the pilgrims to Saudi Arabia, the risk of NTDs is magnified. Therefore, the future plans for Umrah and Hajj expansion also include strengthening the healthcare system and constructing a national plan to deal with potential public health threats. The Saudi government’s health sector expansion also includes research and development (R&D) capacity building. For example, Saudi Arabia intends to build a national center for vaccine and biologic research and development within King Saud University (KSU) in Riyadh. KSU is a leading university in healthcare research, and in order to support establishing this center, it signed an agreement with the Texas Children’s Hospital Center for Vaccine Development, focusing on education and training and to support the transfer of technology to the Kingdom of Saudi Arabia. This collaboration will help to train Saudi scientists at various steps of vaccine development and help to establish a parallel vaccine facility in Saudi Arabia. The ultimate goal for the Saudi’s national center for vaccine and biologic research and development is to help in the research and development of vaccine candidates against local and regional infectious diseases, including major NTDs in the region. Furthermore, the Saudi Ministry of Energy, Industry, and Mineral Resources is sponsoring a new Saudi Vaccine and Biopharmaceutical Center funded by the King Abdulaziz City for Science and Technology (KACST, the major funding agency for R&D in Saudi) and operated by SaudiVax and PnuVax, applying the concept of government-owned, contractor-operated model (GOCO). The center will have the capacity to scale up cell-based vaccines up to 2,000 L under good manufacturing practice (GMP) guidelines with approval from the Saudi Food and Drug Authority (SFDA). The idea of the center is to support moving early discoveries from the bench to the market, especially those with low global market value and so week incentives for large multinationals to support. Finally, the Saudi government has formed a thinktank with representatives from the different groups mentioned above and from the policy-making side that is called the “Saudi Vaccine Alliance.” This thinktank is used to address matters related to combating new issues and to expand on the Saudi reach to the international scientific community.

Similar efforts will be required to expand disease diagnostic capacity. In that context, the Saudi Ministry of Health has recently mandated the expansion of the National Health Laboratory (NHL) located north of Riyadh, with emphasis on increased capacity and scope for its reference diagnostic labs, including three core laboratories: infectious diseases, genetic diseases, and molecular biology systems. The NHL aims to support the Saudi’s strategic plans by achieving high standards in scientific research, diagnosis, and disease prevention. The NHL expansion includes the establishment of a new Center for Neglected Tropical Diseases.

Indeed, during the coming years, we can continue to expect a large influx of pilgrims to Saudi Arabia, especially from countries known to be endemic for NTDs. Therefore, there is an urgent need for development of more comprehensive surveillance programs and data collection not only during the Hajj but also around the year to account for Umrah pilgrims. Such programs could monitor for any possible outbreaks for all NTDs highlighted above, with an emphasis on countries with the highest number of pilgrims coming from NTD-endemic nations. In addition, surveillance monitoring will continue to include Hajj and Umrah pilgrims leaving Saudi Arabia and traveling globally. Collectively, these data will enable the policymakers to make informed decisions to control and prevent NTDs in the Middle East and also in all of the Muslim-majority nations comprising the Organization of the Islamic Conference (OIC).

In parallel, many of the NTDs highlighted above are transmitted by insects and ticks, which are now widespread in the western region of Saudi Arabia. Therefore, intensified vector management and control will represent a critical component of health sector planning for the Hajj and Umrah. Toward that goal, the Saudi Ministry of Health through the Center for NTDs will develop programs to focus on vector controls and surveillances.

In summary, mass gatherings may facilitate the emerging of NTDs just as they do other diseases of epidemic or pandemic potential. Since the Hajj and Umrah represent international gatherings, collaboration at multiple levels is required, including expanded surveillance and intensified vector management and control, increased reference laboratory diagnostic testing, and R&D for both new diagnostics and vaccines. Through its strategic planning, the Ministry of Health and its research university and institute partners are committed to undertaking a multidimensional approach to disease threats in the Kingdom of Saudi Arabia, the MENA region, and across the OIC nations.

## References

[1] U.S. Department of State. Travel. State. Gov. Hajj and Umrah. [March 7, 2018]. Available from: https://travel.state.gov/content/travel/en/international-travel/before-you-go/travelers-with-special-considerations/hajj-umrah.html.

[2] Al-Tawfiq JA, Gautret P, Memish ZA. Expected immunizations and health protection for Hajj and Umrah 2018 -An overview. Travel Med Infect Dis. 2017;19:2–7. Epub 2017/10/19. 10.1016/j.tmaid.2017.10.005 . 29037978 PMC7110709

[3] ILM Feed. ILMFeed.com. Which Countries Have the Highest Number of Hajj Pilgrims? [March 7, 2018]. Available from: http://ilmfeed.com/which-countries-have-the-highest-number-of-hajj-pilgrims/

[4] Herricks JR, Hotez PJ, Wanga V, Coffeng LE, Haagsma JA, Basanez MG, et al The global burden of disease study 2013: What does it mean for the NTDs? PLoS Negl Trop Dis. 2017;11(8):e0005424 10.1371/journal.pntd.0005424 ; PubMed Central PMCID: PMC5542388. 28771480 PMC5542388

[5] Salmon-Rousseau A, Piednoir E, Cattoir V, de La Blanchardiere A. Hajj-associated infections. Med Mal Infect. 2016;46(7):346–54. Epub 2016/05/28. 10.1016/j.medmal.2016.04.002 . 27230822 PMC7131558

[6] Memish ZA, Zumla A, Alhakeem RF, Assiri A, Turkestani A, Al Harby KD, et al Hajj: infectious disease surveillance and control. Lancet. 2014;383(9934):2073–82. Epub 2014/05/27. 10.1016/S0140-6736(14)60381-0 . 24857703 PMC7137990

[7] Kraemer MU, Sinka ME, Duda KA, Mylne A, Shearer FM, Brady OJ, et al The global compendium of Aedes aegypti and Ae. albopictus occurrence. Sci Data. 2015;2:150035 Epub 2015/07/16. 10.1038/sdata.2015.35 ; PubMed Central PMCID: PMC4493829. 26175912 PMC4493829

[8] Bin Ghouth AS, Amarasinghe A, Letson GW. Dengue outbreak in Hadramout, Yemen, 2010: an epidemiological perspective. Am J Trop Med Hyg. 2012;86(6):1072–6. Epub 2012/06/06. 10.4269/ajtmh.2012.11-0723 ; PubMed Central PMCID: PMC3366525. 22665621 PMC3366525

[9] El-Kafrawy SA SS, Ela SA, Abd-Alla AMM, Alhabban R, et al Multiple Introductions of Dengue 2 Virus Strains into Saudi Arabia from 1992 to 2014. Vector-borne Zoonotic Dis. 2016;16:391–9. 10.1089/vbz.2015.1911 27135750 PMC4884338

[10] Al-Saeed MS, El-Kafrawy SA, Farraj SA, Al-Subhi TL, Othman NA, Alsultan A, et al Phylogenetic characterization of circulating Dengue and Alkhumra Hemorrhagic Fever viruses in western Saudi Arabia and lack of evidence of Zika virus in the region: A retrospective study, 2010–2015. J Med Virol. 2017;89(8):1339–46. Epub 2017/02/16. 10.1002/jmv.24785 . 28198548 PMC7167144

[11] Ahmed QA, Kattan RF, Memish ZA. Hajj 2016: Under the shadow of global Zika spread. Am J Infect Control. 2016;44(12):1449–50. Epub 2016/10/30. 10.1016/j.ajic.2016.09.002 . 27751616

[12] Zumla A, McCloskey B, Bin Saeed AA, Dar O, Al Otabi B, Perlmann S, et al What is the experience from previous mass gathering events? Lessons for Zika virus and the Olympics 2016. Int J Infect Dis. 2016;47:1–4. Epub 2016/06/21. 10.1016/j.ijid.2016.06.010 . 27321962 PMC7110488

[13] Ahmed QA, Memish ZA. Yellow fever and Hajj: with all eyes on Zika, a familiar flavivirus remains a threat. Front Med. 2016;10(4):527–30. Epub 2016/10/21. 10.1007/s11684-016-0487-2 . 27757795

[14] Elachola H, Ditekemena J, Zhuo J, Gozzer E, Marchesini P, Rahman M, et al Yellow fever outbreaks, vaccine shortages and the Hajj and Olympics: call for global vigilance. Lancet. 2016;388(10050):1155 Epub 2016/09/10. 10.1016/S0140-6736(16)31546-X . 27609407 PMC7134621

[15] Samy AM, Peterson AT, Hall M. Phylogeography of Rift Valley Fever Virus in Africa and the Arabian Peninsula. PLoS Negl Trop Dis. 2017;11(1):e0005226 Epub 2017/01/10. 10.1371/journal.pntd.0005226 ; PubMed Central PMCID: PMC5221768. 28068340 PMC5221768

[16] Shivanna V, McDowell C, Wilson WC, Richt JA. Complete Genome Sequence of Two Rift Valley Fever Virus Strains Isolated from Outbreaks in Saudi Arabia (2000) and Kenya (2006 to 2007). Genome Announc. 2016;4(5). Epub 2016/09/10. 10.1128/genomeA.00926-16 ; PubMed Central PMCID: PMC5017218. 27609913 PMC5017218

[17] Al-Afaleq AI, Hussein MF. The status of Rift Valley fever in animals in Saudi Arabia: a mini review. Vector Borne Zoonotic Dis. 2011;11(12):1513–20. Epub 2011/09/20. 10.1089/vbz.2010.0245 . 21923257

[18] el-Azazy OM, Scrimgeour EM. Crimean-Congo haemorrhagic fever virus infection in the western province of Saudi Arabia. Trans R Soc Trop Med Hyg. 1997;91(3):275–8. Epub 1997/05/01. . 9231193 10.1016/s0035-9203(97)90072-9

[19] Hassanein KM, El-Azazy OM. Isolation of Crimean-Congo hemorrhagic fever virus from ticks on imported Sudanese sheep in Saudi Arabia. Ann Saudi Med. 2000;20(2):153–4. Epub 2007/02/27. . 17322717 10.5144/0256-4947.2000.153

[20] Zaki AM. Isolation of a flavivirus related to the tick-borne encephalitis complex from human cases in Saudi Arabia. Trans R Soc Trop Med Hyg. 1997;91(2):179–81. Epub 1997/03/01. . 9196762 10.1016/s0035-9203(97)90215-7

[21] Charrel RN, Zaki AM, Fakeeh M, Yousef AI, de Chesse R, Attoui H, et al Low diversity of Alkhurma hemorrhagic fever virus, Saudi Arabia, 1994–1999. Emerg Infect Dis. 2005;11(5):683–8. Epub 2005/05/14. 10.3201/eid1105.041298 ; PubMed Central PMCID: PMC3320364. 15890119 PMC3320364

[22] http://kingabdullahfellowship.com/wp-content/uploads/ZahraPoster-Final-4-27-16.pdf.

[23] Al-Tawfiq JA, Memish ZA. Mass gathering medicine: 2014 Hajj and Umra preparation as a leading example. Int J Infect Dis. 2014;27:26–31. Epub 2014/08/17. 10.1016/j.ijid.2014.07.001 . 25128639 PMC7110515

[24] Memish ZA, Al-Tawfiq JA. The Hajj in the time of an Ebola outbreak in West Africa. Travel Med Infect Dis. 2014;12(5):415–7. Epub 2014/09/27. 10.1016/j.tmaid.2014.09.003 . 25257580 PMC7128612

[25] Widagdo W, Okba NMA, Stalin Raj V, Haagmans BL. MERS-coronavirus: From discovery to intervention. One Health. 2017;3:11–6. Epub 2017/06/16. 10.1016/j.onehlt.2016.12.001 ; PubMed Central PMCID: PMC5454172. 28616497 PMC5454172

[26] Abuzaid AA, Abdoon AM, Aldahan MA, Alzahrani AG, Alhakeem RF, Asiri AM, et al Cutaneous Leishmaniasis in Saudi Arabia: A Comprehensive Overview. Vector Borne Zoonotic Dis. 2017;17(10):673–84. Epub 2017/08/15. 10.1089/vbz.2017.2119 ; PubMed Central PMCID: PMC5649416. 28806141 PMC5649416

[27] Salam N, Al-Shaqha WM, Azzi A. Leishmaniasis in the middle East: incidence and epidemiology. PLoS Negl Trop Dis. 2014;8(10):e3208 Epub 2014/10/03. 10.1371/journal.pntd.0003208 ; PubMed Central PMCID: PMC4183486. 25275483 PMC4183486

[28] El Hajj L, Thellier M, Carriere J, Bricaire F, Danis M, Caumes E. Localized cutaneous leishmaniasis imported into Paris: a review of 39 cases. Int J Dermatol. 2004;43(2):120–5. Epub 2004/05/06. . 15125502 10.1111/j.1365-4632.2004.01991.x

[29] Yun TY, Eun HC, Lee YS, Chi JG, Ham EK, Hong ST, et al Two cases of imported cutaneous leishmaniasis in Korea. Kisaengchunghak Chapchi. 1985;23(2):327–30. Epub 1985/12/01. . 12888678 10.3347/kjp.1985.23.2.327

[30] Zhang M, Liu F, Liu H, Hu W, Sang H. Imported cutaneous leishmaniasis caused by Leishmania major in a Chinese laborer who worked in Saudi Arabia. An Bras Dermatol. 2016;91(3):365–7. Epub 2016/07/22. 10.1590/abd1806-4841.20163820 ; PubMed Central PMCID: PMC4938285. 27438208 PMC4938285

[31] Bailey F, Mondragon-Shem K, Hotez P, Ruiz-Postigo JA, Al-Salem W, Acosta-Serrano A, et al A new perspective on cutaneous leishmaniasis-Implications for global prevalence and burden of disease estimates. PLoS Negl Trop Dis. 2017;11(8):e0005739 Epub 2017/08/11. 10.1371/journal.pntd.0005739 ; PubMed Central PMCID: PMC5552022. 28796782 PMC5552022

[32] Du R, Hotez PJ, Al-Salem WS, Acosta-Serrano A. Old World Cutaneous Leishmaniasis and Refugee Crises in the Middle East and North Africa. PLoS Negl Trop Dis. 2016;10(5):e0004545 Epub 2016/05/27. 10.1371/journal.pntd.0004545 ; PubMed Central PMCID: PMC4882064. 27227772 PMC4882064

[33] World Health Organization. World malaria report 2017 [March 8, 2018]. Available from: http://www.who.int/malaria/publications/world-malaria-report-2017/report/en/.

[34] Memish ZA, Alzahrani M, Alhakeem RF, Bamgboye EA, Smadi HN. Toward malaria eradication in Saudi Arabia: evidence from 4-year surveillance in Makkah. Ann Saudi Med. 2014;34(2):153–8. Epub 2014/06/05. 10.5144/0256-4947.2014.153 . 24894785 PMC6074865

[35] Khan AS, Qureshi F, Shah AH, Malik SA. Spectrum of malaria in Hajj pilgrims in the year 2000. J Ayub Med Coll Abbottabad. 2002;14(4):19–21. Epub 2003/04/12. . 12688096

[36] Abd El Ghany M, Alsomali M, Almasri M, Padron Regalado E, Naeem R, Tukestani A, et al Enteric Infections Circulating during Hajj Seasons, 2011–2013. Emerg Infect Dis. 2017;23(10). Epub 2017/09/21. 10.3201/eid2310.161642 ; PubMed Central PMCID: PMC5621540. 28930004 PMC5621540

[37] Olaitan AO, Dia NM, Gautret P, Benkouiten S, Belhouchat K, Drali T, et al Acquisition of extended-spectrum cephalosporin- and colistin-resistant Salmonella enterica subsp. enterica serotype Newport by pilgrims during Hajj. Int J Antimicrob Agents. 2015;45(6):600–4. Epub 2015/03/15. 10.1016/j.ijantimicag.2015.01.010 . 25769786

[38] Amani S. Alqahtani MA, Paul Arbond, Robert Booy, Harunor Rashid. Burden of vaccine preventable diseases at large events. Vaccine. 2015;33:6552–63. 10.1016/j.vaccine.2015.09.076 26437018

[39] Alimuddin Zumla BM, Tina Endericks, Esam I Azhar EP. The challenges of cholera at the 2017 Hajj pilgrimage. Lancet Infect Dis. 2017;17(9):895–7. 10.1016/S1473-3099(17)30454-1 28803813 PMC7129522

[40] Kingdom of Saudi Arabia. Vision 2030. National Transformation Program [March 7, 2018]. Available from: http://vision2030.gov.sa/en/ntp.

